# Impact of continuous glucose monitoring on glycaemic risk index in adults with type 1 diabetes using multiple daily insulin injections in the GOLD trial

**DOI:** 10.3389/fcdhc.2026.1767987

**Published:** 2026-03-04

**Authors:** Daniel Pylov, Sofia Sterner Isaksson, Henrik Imberg, David Klonoff, Marcus Lind

**Affiliations:** 1 Department of Molecular and Clinical Medicine, Institute of Medicine, Sahlgrenska Academy, University of Gothenburg, Gothenburg, Sweden; 2 Department of Medicine, NU Hospital Group, Uddevalla, Sweden; 3 Statistiska Konsultgruppen Sweden AB, Gothenburg, Sweden; 4 Diabetes Research Institute, Mills-Peninsula Medical Center, San Mateo, CA, United States; 5 Department of Medicine, Geriatrics and Emergency Care, Sahlgrenska University Hospital, Gothenburg, Sweden

**Keywords:** continuous glucose monitoring, glycaemia risk index, participant-reported outcomes, self-monitoring of blood glucose, time in range

## Abstract

**Background:**

Continuous glucose monitoring (CGM) has significantly improved glycaemic management in individuals with diabetes. The Glycaemia Risk Index (GRI) is a composite metric based on CGM data that provides a comprehensive evaluation of glycaemic quality, incorporating both hypoglycaemia and hyperglycaemia. This study evaluated the impact of transitioning from self-monitoring of blood glucose (SMBG) to CGM on GRI in adults with type 1 diabetes (T1D) using multiple daily injection (MDI) insulin therapy.

**Methods:**

Secondary analyses were conducted in 125 adults with T1D from the randomised GOLD trial. Participants alternated between CGM and SMBG for two 26-week periods, separated by a 17-week wash-out. The GRI was calculated on a 0–100 scale from CGM data and categorised into five risk zones. Associations between baseline characteristics and participant-reported outcomes such as diabetes-related behaviours, lifestyle, psychological characteristics, and changes in GRI were also explored.

**Results:**

Transitioning from SMBG to CGM significantly reduced the overall GRI by 9.8 units (95% CI −13.3, −6.3), with decreases in both hypoglycaemia (–1.8, 95% CI −2.4, −1.2) and hyperglycaemia (–2.8, 95% CI −5.3, −0.4) components. GRI zone classification was maintained or improved in 85.4% (105/123, *P* <.001) of participants. The GRI correlated moderately with TIR (*r* = –0.47, 95% CI −0.60, −0.32), but standardised effect sizes were larger for GRI than for TIR (–0.5 [95% CI –0.72, –0.34] vs. 0.2 [95% CI 0.00, 0.37]). Exploratory analyses suggested that self-reported psychosocial traits influenced GRI changes: thoroughness was linked to greater reductions in hypoglycaemia risk, whereas distractibility, self-described laziness, and carbohydrate counting training were associated with smaller improvements.

**Conclusion:**

Switching from SMBG to CGM significantly improved GRI in adults with T1D on MDI therapy. Compared with TIR, GRI demonstrated greater responsiveness to treatment-related changes. As a composite metric that integrates both hypo- and hyperglycaemia, GRI may serve as a valuable endpoint for evaluating interventions and as a complementary measure in clinical practice.

## 1 Introduction

The management of type 1 diabetes (T1D) is a dynamic and evolving field. Glycated haemoglobin (HbA1c) has long been the standard measure of long-term glycaemic control and remains the gold-standard biomarker for assessing the risk of diabetes-related complications (1–3). However, in some individuals, HbA1c does not accurately reflect mean glucose levels (4), which may lead to suboptimal treatment decisions and potential under- or overtreatment (5). Moreover, HbA1c represents only an average value over a prolonged period, limiting its usefulness when detailed glucose patterns are required for individualised management (6).

In recent years, continuous glucose monitoring (CGM) has become increasingly common in T1D care, enabling more detailed assessments of glucose patterns (7, 8). CGM data are typically summarised in the Ambulatory Glucose Profile (AGP) report (9), which presents the means of eight CGM metrics (although two of these, mean glucose and glucose management indicator [GMI] are linearly related), including time in range (TIR). While the AGP report is a valuable tool, the presentation of multiple metrics can be challenging to interpret, particularly when some metrics improve while others deteriorate.

TIR provides a straightforward summary of overall glycaemic exposure, accounting for both low and high glucose levels. However, TIR alone does not capture the frequency or severity of glycaemic excursions (10, 11), nor does it incorporate variability, and should therefore be interpreted alongside complementary metrics such as time below range (TBR) and time above range (TAR) (8). To address these limitations, composite metrics have been proposed to provide a more holistic evaluation of glycaemic control (11–13). Nevertheless, such metrics may be challenging to implement in clinical practice, particularly in primary care settings where clinicians must manage multiple responsibilities and monitor a wide range of health indicators beyond glucose control.

The Glycaemic Risk Index (GRI) (14) provides a single numerical summary of glycaemic quality, derived from expert evaluations of the risks associated with hypo- and hyperglycaemia. It is based on time spent in very low (<3.0 mmol/L), low (3.0–3.9 mmol/L), high (10.0–13.9 mmol/L), and very high (≥13.9 mmol/L) glucose ranges, with greater weight assigned to more pronounced deviations and to hypoglycaemia than to hyperglycaemia (14). By emphasising more pronounced hypo- and hyperglycaemia, the GRI may offer a more clinically relevant assessment of glycaemic risk, particularly in individuals experiencing frequent or severe excursions.

In the GOLD trial, we previously demonstrated that transitioning from self-monitoring of blood glucose (SMBG) to CGM improved HbA1c, TIR, treatment satisfaction, hypoglycaemia confidence, and well-being in adults with T1D using multiple daily insulin injection (MDI) therapy (15, 16). Further analyses showed that individuals with higher baseline HbA1c, greater TAR stage 2, lower TBR stage 1 and 2, and lower coefficient of variation (CV) experienced greater HbA1c reductions after switching to CGM (17). However, conventional metrics such as HbA1c and standard CGM parameters demonstrated limited associations with improvements in participant-reported outcomes (17) and only marginally explained the improvements in well-being (18). Given its broader dynamic range and weighting of more pronounced glucose values, the GRI may be more sensitive in capturing such associations.

The aim of this study was to evaluate the effects of switching from SMBG to CGM on GRI in adults with T1D using MDI therapy, and the associations between participant-reported outcomes and GRI.

## 2 Materials and methods

### 2.1 Study design

This is a secondary analysis based on data from the GOLD trial. The design of the trial has been described in detail previously (15, 19). Briefly, it was an open-label, randomised clinical trial with a crossover design, conducted in Sweden between 2014 and 2016. The trial was approved by the regional ethics committee in Gothenburg (No. 857-13) and was registered on ClinicalTrials.gov (NCT02092051).

Before randomisation, participants underwent a run-in period of up to six weeks. They were then randomly assigned to either CGM-guided treatment using real-time CGM for 26 weeks or SMBG-guided treatment. After a 17-week wash-out period, participants switched to the alternate treatment arm. During the SMBG phase, masked CGM was used during two of the final four weeks to collect data on hypoglycaemia, hyperglycaemia, TIR, and glycaemic variability. Participants could not view their CGM data during this phase, but the data were collected for comparison with the active CGM phase. CGM and SMBG data were regularly downloaded throughout the study.

### 2.2 Outcomes

Participant reported outcomes (PROs) were measured at baseline and before and after each intervention phase (CGM or SMBG). The following questionnaires were used:

the Diabetes Treatment Satisfaction Questionnaire, status version (DTSQs) and change version (DTSQc) (20, 21); the Hypoglycaemia Confidence Scale (HCS) (22); the Swedish Hypoglycaemia Fear Survey (Swe-HFS) (23); Problem Areas in Diabetes, Swedish version (Swe-PAID-20) (24); and the WHO-5 Well-Being Index (25). In addition, a study-specific questionnaire (PredQ) was used to evaluate participants’ diabetes-related behaviours, lifestyle, and psychological factors that could influence glycaemic control.

The GRI was evaluated based on masked CGM data from the end of each treatment phase. It is derived from weighted contributions of time spent in very low (<3.0 mmol/L), low (3.0–3.9 mmol/L), high (10.0–13.9 mmol/L), and very high (≥13.9 mmol/L) glucose ranges. The components are calculated as follows:


Hypoglycaemia component=(very low)+0.8×(low)

Hyperglycaemia component=(very high)+0.5×(high)

The overall score is then obtained as: GRI = (3.0 × hypoglycaemia component) + (1.6 × hyperglycaemia component), capped at a maximum value of 100 (14).

The GRI was evaluated as a composite outcome, with separate analyses of the hypoglycaemia and hyperglycaemia components to assess whether CGM had differential effects on each of the two domains of glycaemic risk. The two components were also visualised using the “GRI grid,” a two-dimensional plot with hypoglycaemia component on the horizontal axis and hyperglycaemia component on the vertical axis. The grid is divided into five zones (A–E) in 20-point GRI-increments, where Zone A represents the most favourable and Zone E the least favourable overall profile (14).

### 2.3 Analyses

We evaluated differences in the GRI total score and in the hypoglycaemia and hyperglycaemia components between the CGM and SMBG phases. Standardised effect sizes were calculated for GRI and other CGM metrics, including mean glucose, TIR, TBR level 1 (<3.9 mmol/L), TBR level 2 (<3.0 mmol/L), TAR level 1 (>10.0 mmol/L), and TAR level 2 (>13.9 mmol/L). In addition, we conducted correlation analyses between GRI and other CGM variables, as well as exploratory analyses to identify potential baseline predictors for changes in GRI, including age, sex, and PRO measures.

### 2.4 Statistical methods

Continuous variables were summarised using means and standard deviations (SDs) or medians with interquartile ranges (IQRs), as appropriate, and categorical variables using counts and percentages.

Differences in the GRI total score and its hypoglycaemia and hyperglycaemia components between the CGM and SMBG phases were analysed using linear mixed-effects models, with treatment and study period as fixed effects and participant as a random effect, as appropriate for crossover trials. Robust standard errors (HC3 method) were used to account for possible deviations from model assumptions. Standardised effect sizes (Cohen’s *d*) were calculated as the mean difference divided by the pooled SD and interpreted as small (≈0.2), medium (≈0.5), or large (≈0.8) (26).

Correlation analyses were performed using Pearson correlation coefficients, with adjustment for baseline values using partial correlations. For binary variables, differences in changes in GRI were evaluated using two-sample *t* tests comparing mean changes in GRI between groups.

All tests were two-tailed and conducted at the 5% significance level, with corresponding 95% confidence intervals. Statistical analyses were performed using SAS/STAT^®^ software, version 9.4 (SAS Institute Inc. Cary, NC, USA).

## 3 Results

### 3.1 Participant characteristics

Of the 161 participants randomised, 19 were excluded from the full analysis set because they had no follow-up data in one of the treatment periods. An additional 17 participants lacked raw CGM data in both treatment phases, which were required for GRI evaluation, and were therefore excluded from the present analyses.

The baseline characteristics of the 125 participants included in the present analysis were as follows: mean (SD) age was 44.8 (12.8) years, 54 (43.2%) were women, mean duration of T1D was 22.3 (11.8) years, and mean HbA1c was 68.8 (8.8) mmol/mol or 8.4% (0.8%). The median (IQR) number of days with masked CGM was 13.0 (11.7–13.6). At baseline, the median number of self-reported hypoglycaemic episodes was 2.0 (IQR 1.0–2.5) per week over the preceding two months. Severe hypoglycaemia during the past year was reported by 5 participants (4.0%). The mean (SD) baseline GRI score was 70.0 (17.4), with 37 participants (30.6%) in zone E, 52 (43.0%) in zone D, 26 (21.5%) in zone C, 6 (5.0%) in zone B, and none in zone A. Overall, these characteristics were similar to those of all randomised participants (n=161) (**Table 1**).

**Table 1:** Baseline demographics and clinical characteristics of participants included in the present analysis and of all randomised participants.

Characteristic	Included in present analysis* (n=125)	All randomised participants (n=161)
Demographics		
Age (years)	44.8 (12.8)	43.7 (13.5)
Female sex, n (%)	54 (43.2%)	73 (45.3%)
Current or previous smoker, n (%)	40 (32.0%)	57 (35.4%)
Diabetes duration (years)	22.3 (11.8)	22.3 (12.0)
HbA1c (mmol/mol)	68.8 (8.8)	70.2 (10.4)
HbA1c (%)	8.4 (0.8)	8.6 (1.0)
Treatment satisfaction and quality of life		
DTSQs total score †	25.6 (5.8)	25.3 (5.8)
WHO-5 Well-Being Index ‡	60.4 (17.8)	60.5 (17.4)
Swe-HFS: Behaviour/Avoidance mean score §	1.9 (0.6)	1.9 (0.6)
Swe-HFS: Worry mean score §	0.8 (0.7)	0.9 (0.7)
SWE-PAID 20 total scale ‖	25.2 (16.8)	26.0 (17.8)
HCS total score ¶	3.3 (0.5)	3.2 (0.5)
Glucose metrics		
Mean glucose level (mmol/L)	10.8 (1.7)	10.8 (1.8)
Time below range, level 1 (<3.9 mmol/L) (%)	5.5 (4.3)	5.6 (4.4)
Time below range, level 2 (<3.0 mmol/L) (%)	2.2 (2.4)	2.2 (2.4)
Time in range (3.9–10.0 mmol/L) (%)	41.5 (12.8)	41.6 (12.7)
Time above range, level 1 (>10.0 mmol/L) (%)	53.0 (15.0)	52.8 (14.9)
Time above range, level 2 (>13.9 mmol/L) (%)	25.6 (13.3)	25.3 (13.3)
Self-reported hypoglycaemia history		
Hypoglycaemia episodes past 2 months (no./week), median (IQR)	2.0 (1.0–2.5)	2.0 (1.0–2.5)
Any severe hypoglycaemia past year, n (%)	5 (4.0%)	12 (7.5%)
Any severe hypoglycaemia past 5 years, n (%)	29 (23.2%)	40 (24.8%)
Glycaemia Risk Index (GRI)**		
GRI total score	70.0 (17.4)	69.8 (17.4)
Hypoglycaemia component	4.5 (3.7)	4.6 (3.7)
Hyperglycaemia component	35.4 (12.3)	35.2 (12.2)
GRI zone, n (%)**		
A	0 (0.0%)	0 (0.0%)
B	6 (5.0%)	6 (4.8%)
C	26 (21.5%)	28 (22.6%)
D	52 (43.0%)	52 (41.9%)
E	37 (30.6%)	38 (30.6%)

Continuous variables are presented as mean (SD) unless otherwise specified; categorical variables are presented as number (percentage).

* Current analysis based on the full analysis set (FAS), restricted to participants with available masked CGM data in at least one of the treatment periods.

† DTSQs (Diabetes Treatment Satisfaction Questionnaire, status version); score range 0–36. Higher scores indicate greater treatment satisfaction.

‡ WHO-5 Well-Being Index; score range 0–100, calculated from raw scores (0–25) × 4. Higher scores indicate better emotional well-being.

§ Swe-HFS (Swedish Hypoglycaemic Fear Survey); mean item score range 0–4; higher scores reflect more frequent avoidance behaviours (Behaviour/Avoidance subscale) and greater anxiety related to hypoglycaemia (Worry subscale).

‖ Swe-PAID-20 (Swedish version of the Problem Areas in Diabetes scale); total score range 0–80. Higher scores indicate greater diabetes-related emotional distress.

¶ HCS (Hypoglycaemic Confidence Scale); mean item score range 1–4. Higher scores reflect greater confidence in managing hypoglycaemia.

** Baseline GRI values were available for 121 of 125 participants in the present analysis and for 124 of 161 randomised participants.

DTSQs, Diabetes Treatment Satisfaction Questionnaire; GRI, Glycaemia Risk Index; HCS, Hypoglycaemic Confidence Scale; HFS, Hypoglycaemia Fear Survey; IQR, interquartile range; PAID, Problem Areas in Diabetes; SD, standard deviation; TAR, Time Above Range; TBR, Time Below Range; TIR, Time In Range; WHO-5, World Health Organization–5 Well-Being Index.

### 3.2 Effects on GRI with CGM compared to SMBG

Transitioning from SMBG to CGM reduced the overall GRI by 9.8 units (95% CI −13.3, −6.3, *P* <.001). The hypoglycaemia component decreased by 1.8 units (−2.4, −1.2, *P* <.001) and the hyperglycaemia component by 2.8 units (−5.3, −0.4, *P* = .025) (**Table 2**).

**Table 2:** Glycaemia Risk Index (GRI) scores during continuous glucose monitoring (CGM) compared to self-monitoring of blood glucose (SMBG).

GRI measure	CGM (n=123)	SMBG (n=125)	Mean difference (95% CI)	*P*
GRI total score	57.7 (19.3)	67.3 (17.7)	−9.8 (−13.3, −6.3)	<.001
Hypoglycaemia component	2.4 (2.6)	4.2 (3.6)	−1.8 (−2.4, −1.2)	<.001
Hyperglycaemia component	31.7 (13.6)	34.4 (12.8)	−2.8 (−5.3, −0.4)	0.025

Results are presented as estimated marginal means (EMMEANS) and standard deviations (SD), mean difference with 95% confidence intervals (CI), and effect size (Cohen *d*) calculated as the mean difference divided by the pooled SD.

Statistical analyses were performed using linear mixed-effects models for crossover trials, with treatment and study period as fixed effects and participant as a random effect. Robust standard errors (HC3 method) were used to account for potential deviations from distributional assumptions.

CGM, Continuous Glucose Monitoring; CI, confidence interval; GRI, Glycaemia Risk Index; SD, standard deviation; SMBG, Self-Monitoring of Blood Glucose.

**Figure 1:**
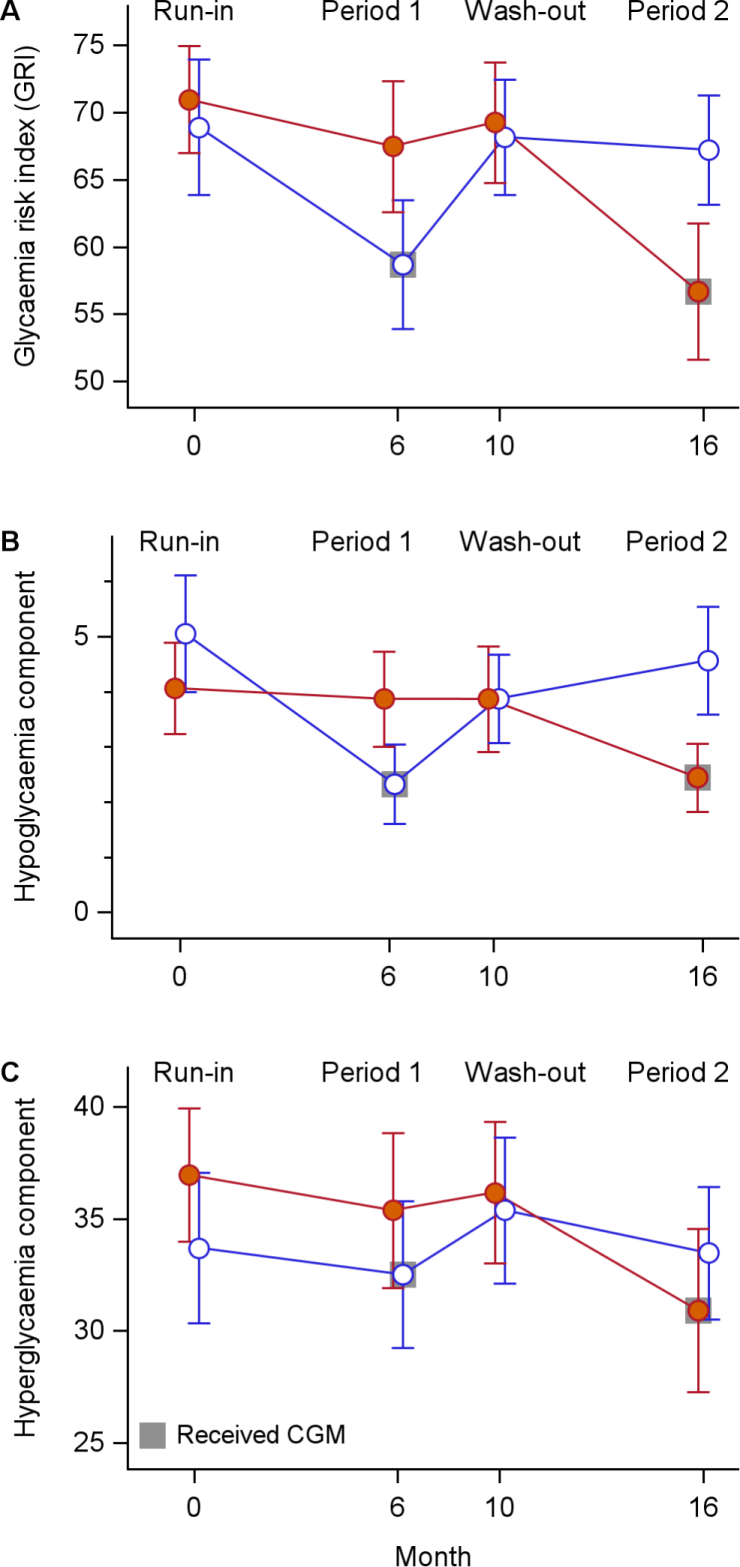
Longitudinal changes in Glycaemia Risk Index [GRI; **(A)**], hypoglycaemia component **(B)**, and hyperglycaemia component **(C)** during the study period. Points represent mean values, and vertical lines indicate 95% confidence intervals. Red filled circles indicate participants who received SMBG first; blue open circles indicate participants who received CGM first. Grey squares indicate the period during which participants received CGM.

**Figure 2:**
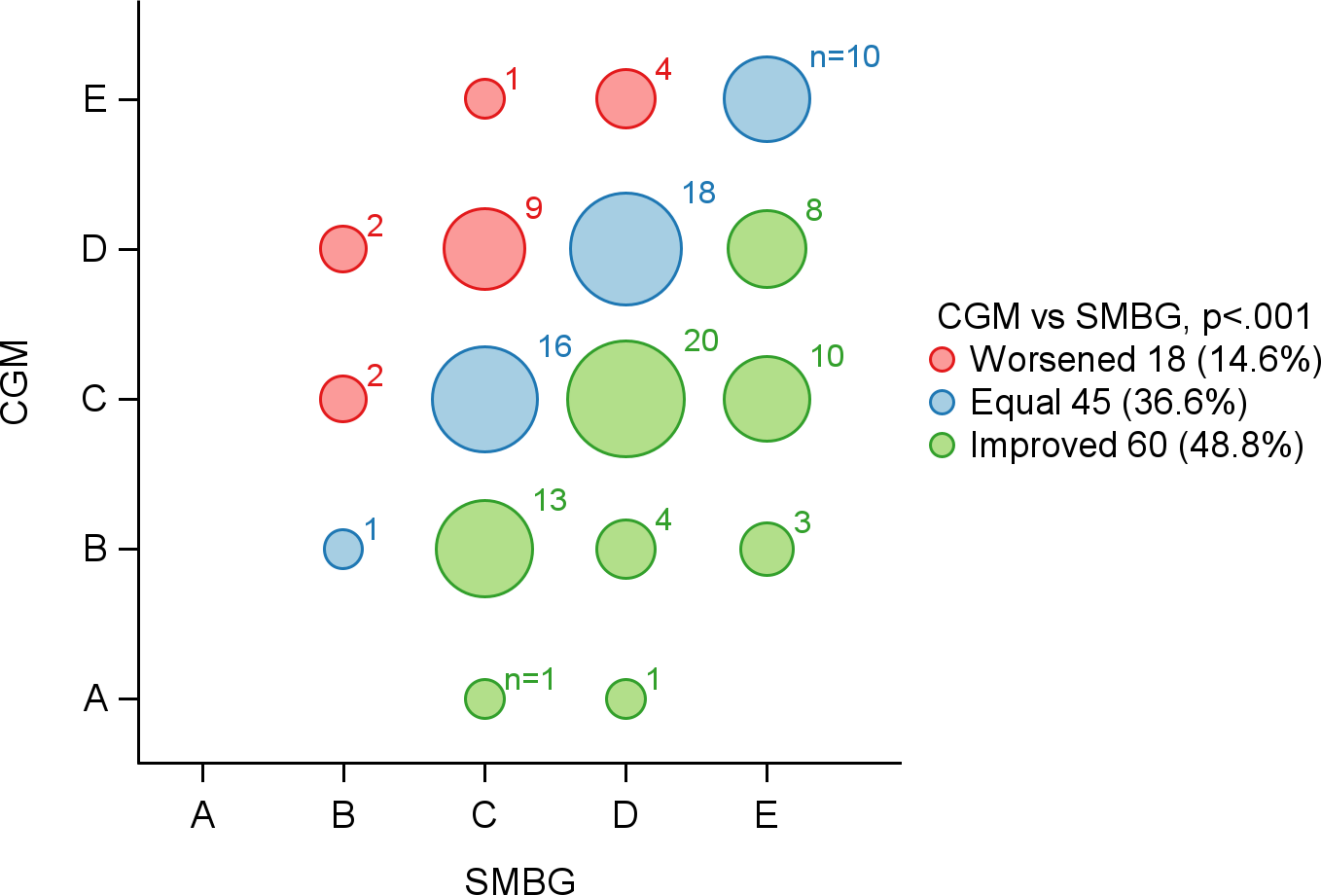
Change in Glycaemia Risk Index (GRI) zones (A–E) between continuous glucose monitoring (CGM) and self-monitoring of blood glucose (SMBG). Each bubble represents the number of participants who moved from one GRI zone during SMBG (x-axis) to another during CGM (y-axis), with bubble size proportional to the number of individuals and colours indicating change: green for improvement, blue for no change, and red for worsening. GRI zone A reflects the lowest glycaemic risk and zone E the highest. The majority of participants (85%) either remained in the same zone or improved with CGM compared to SMBG (*P* <.001). Analysis restricted to participants with CGM data available in both treatment periods (n=123).

**Figure 3:**
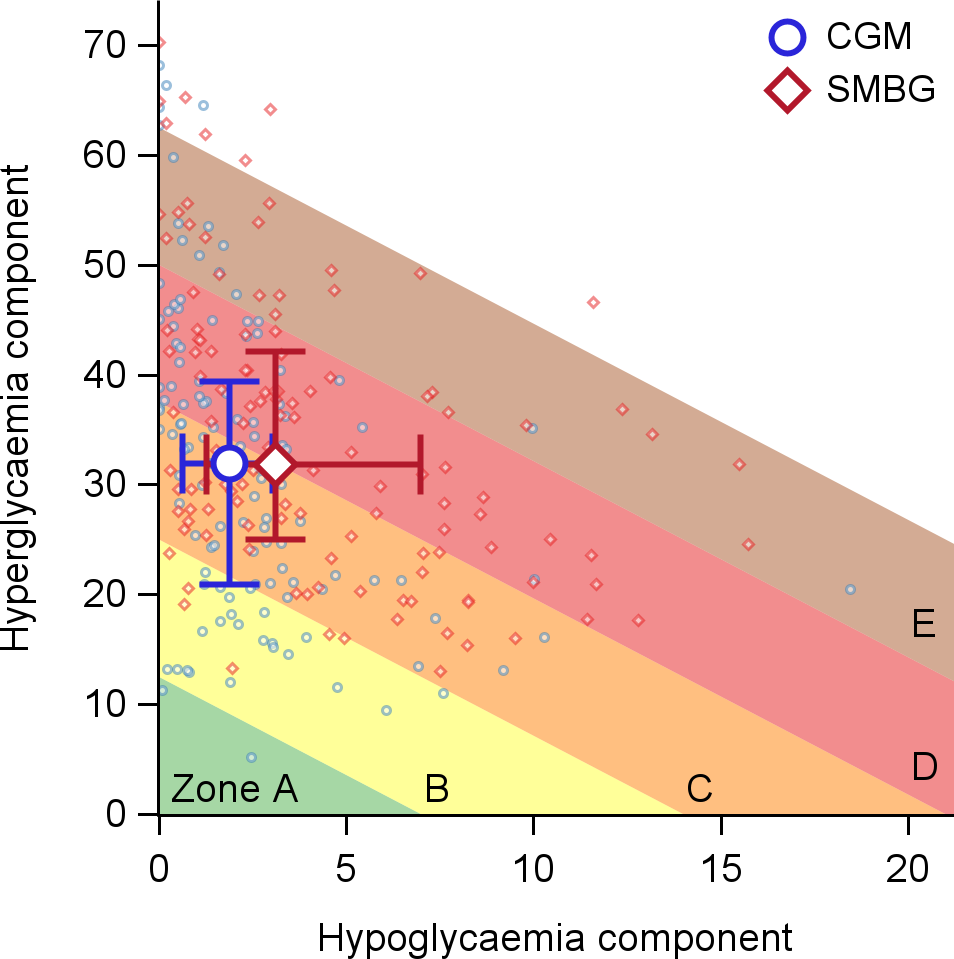
Glycaemia Risk Index (GRI) profiles during continuous glucose monitoring (CGM) and self-monitoring of blood glucose (SMBG). Each point represents an individual’s hypoglycaemia and hyperglycaemia component scores, with blue circles for CGM and red diamonds for SMBG. Larger open symbols with horizontal and vertical lines indicate group means with 95% confidence intervals. The background is divided into five risk zones (A–E), where Zone A (green) denotes optimal glycaemic control and Zone E (brown) represents the highest risk, based on diagonal thresholds of the composite GRI score. Lower values on both axes reflect better glycaemic quality, and movement toward the lower-left corner indicates improved overall control. Participants generally showed a shift into lower-risk zones during CGM use compared to SMBG.

As illustrated in **Figure 1**, GRI values declined during CGM use irrespective of treatment sequence, with the largest changes observed in the hypoglycaemia component. Consistent with this pattern, 105 of 123 participants (85.4%) with available CGM data in both phases improved or maintained their GRI zone classification during CGM compared with SMBG (P <.001; **Figure 2**), most commonly due to reductions in hypoglycaemia risk. **Figure 3** further demonstrates a general shift of individual GRI profiles toward lower-risk regions during CGM, primarily along the hypoglycaemia axis, with smaller but consistent improvements in hyperglycaemia risk.

### 3.3 Effect sizes for GRI and other CGM metrics

GRI demonstrated a medium effect (–0.53; 95% CI –0.72, –0.34), driven by the hypoglycaemia component (–0.58; –0.77, –0.38), while the hyperglycaemia component showed a small effect (–0.21; –0.40, –0.03). Other CGM metrics showed small to medium effects: mean glucose –0.15 (–0.33, 0.03), TBR level 1 –0.57 (–0.76, –0.37), TBR level 2 –0.64 (–0.84, –0.43), TIR 0.19 (0.00, 0.37), TAR level 1 –0.14 (–0.33, 0.04), and TAR level 2 –0.28 (–0.47, –0.09).

### 3.4 Associations between changes in GRI, its components, and other CGM metrics

Changes in GRI when transitioning from SMBG to CGM were negatively correlated with changes in TIR (*r* = –0.47; 95% CI –0.60, –0.32; *P* <.001) and positively correlated with changes in mean glucose (*r* = 0.65; 0.54, 0.74; *P* <.001), TAR level 1 (*r* = 0.85; 0.79, 0.89; *P* <.001), and TAR level 2 (*r* = 0.83; 0.76, 0.87; *P* <.001). Correlations with changes in TBR level 1 (*r* = 0.11; –0.07, 0.28; *P* = .24) were weak and non-significant, while the association with changes in TBR level 2 was small but significant (*r* = 0.27; 0.10, 0.43; *P* = .002). Changes in GRI were positively correlated with changes in glycaemic variability, measured as the SD of glucose values (*r* = 0.55; 0.41, 0.66; *P* <.001). For comparison, the correlation between changes in TIR and changes in glucose SD was smaller (*r* = −0.34; −0.49, −0.18; *P* <.001).

The hypoglycaemia component was strongly positively correlated with TBR levels 1 and 2, while showing negative correlations with mean glucose and TAR levels 1 and 2, and a moderate positive correlation with TIR. In contrast, the hyperglycaemia component correlated strongly positively with mean glucose and TAR levels 1 and 2, and negatively with TBR levels 1 and 2 and with TIR. Detailed results of all associations are presented in **Supplementary Table 1**.

### 3.5 Associations between changes in GRI and baseline PredQ traits

A greater reduction in the GRI hypoglycaemia component when transitioning from SMBG to CGM was observed among individuals who described themselves as *thorough* (*r* = –0.20; 95% CI –0.36, –0.02, *P* = .030). In contrast, increases in the hypoglycaemia component were found among those who reported being *easily distracted* (*r* = 0.21; 0.03, 0.37, *P* = .024), *lazy* (*r* = 0.27; 0.09, 0.43, *P* = .003), or who had received carbohydrate counting training (*r* = 0.11; –0.10, 0.31, *P* = .041). In contrast, reporting being *lazy* was associated with decreases in the hyperglycaemia component (*r* = –0.21; –0.37, –0.03, *P* = .021). No associations were observed with the GRI total score (**Supplementary Table 2**).

### 3.6 Associations between changes in GRI, its components, and other baseline variables

A greater overall improvement in GRI during CGM compared with SMBG was associated with higher baseline scores on the Hypoglycaemia Fear Survey (HFS) Behaviour/Avoidance scale (*r* = 0.20, 95% CI 0.03, 0.37, *P* = .023) with a similar trend in the HFS/Worry scale (*r* = 0.16; –0.02, 0.33, *P* = .078). A similar relationship was observed for improvements in the hyperglycaemia component of the HFS Behaviour/Avoidance scale (*r* = 0.20; 0.02, 0.37, *P* = .026) (**Supplementary Table 3**).

Smaller reductions in the hypoglycaemia component were associated with higher baseline HbA1c (r = 0.21; 95% CI 0.03, 0.37, *P* = .021), higher baseline mean glucose (r = 0.23; 0.06, 0.39, *P* = .009), and greater time in TAR level 1 (r = 0.21; 0.03, 0.37, *P* = .022). In contrast, greater reductions were observed in individuals with longer diabetes duration (r = −0.29; −0.44, −0.11, *P* = .001), greater time in TBR level 2 (r = −0.44; −0.57, −0.28, *P* < .001) and TBR level 1 (r = −0.41; −0.55, −0.25, *P* < .001), as well as higher baseline scores on the Hypoglycaemia Confidence Scale (r = −0.24; −0.40, −0.06, *P* = .010) (**Supplementary Table 3**). Reductions were also greater among women than men (mean difference −1.4; 95% CI −2.7 to −0.2; *P* = .020). After adjustments for baseline values of GRI, most analyses remained directionally consistent, although some were no longer significant (sex, HbA1c, mean glucose, TBR and TAR; see **Supplementary Table 3**).

No significant correlations between change in GRI scores when transitioning from SMBG to CGM were observed with other baseline variables, including quality of life, well-being, hypoglycaemia confidence, or treatment satisfaction (**Supplementary Table 3**). No significant correlations were observed between changes in GRI and changes in PROs (**Supplementary Table 4**).

## 4 Discussion

Analyses from the GOLD randomised trial demonstrate that transitioning from SMBG to CGM significantly improved GRI in adults with T1D. Both the total score and its hypo- and hyper- glycaemia components decreased, with the most pronounced reduction in hypoglycaemia risk. The standardised effect size (mean difference divided by the SD) for GRI was more than twice that of TIR, likely reflecting the additional impact of the intervention on hypoglycaemia. Greater improvements in GRI were observed among females and in individuals with longer diabetes duration, lower HbA1c, and more baseline hypoglycaemia. Baseline behavioural and psychological characteristics further appeared to modulate the impact of CGM on GRI.

The results align with previous GOLD trial results showing CGM benefits for HbA1c and other CGM metrics (15), and that CGM more effectively reduces hypoglycaemia than hyperglycaemia (16). An earlier study has also shown that GRI more effectively reflects hypoglycaemia than TIR among individuals with diabetes (27). GRI’s relatively strong negative correlation with TIR (*r* = –0.47) confirms its validity, but this corresponds to an explained variance of only about 22%. In other words, more than three-quarters of the variation in GRI reflects aspects of glycaemic control not captured by TIR. This includes the fact that TIR does not distinguish hypo- from hyperglycaemia. The same TIR can exist with a large amount of hypoglycaemia or no hypoglycaemia. Further, the GRI hypoglycaemia component assigns greater weight to more severe or prolonged episodes (<3.0 mmol/L).

Our study also showed that individuals with identical TIR values may have markedly different GRI scores depending on TBR and TAR. This supports earlier observations that TIR alone may not fully capture risk and needs to be interpreted together with TBR and TAR (4, 8). The combination of TIR and TBR offers a clear and intuitive overview of glycaemic control, which remains central in clinical care. While exposure to hypoglycaemia and hyperglycaemia can be individually calculated to evaluate the two domains separately, GRI integrates them both into a single composite score, weighting extreme deviations more heavily. This integration may explain its greater sensitivity to CGM-related changes.

Previous research has linked higher GRI to greater glycaemic variability (28). Since both TIR and coefficient of variation (CV) are associated with vascular complications (29–31), GRI may similarly reflect long-term risk, although this requires further research. In the present study, changes in GRI also correlated more strongly with glycaemic variability than did changes in TIR, suggesting that GRI captures dynamic aspects of glucose fluctuations beyond overall time in range. In this study, the hyperglycaemia component emerged as the main barrier to improved overall glycaemic quality. The GRI Grid’s two-dimensional display may help clinicians quickly determine whether poor glycaemic control is driven mainly by low or high glucose excursions, supporting more targeted treatment adjustments. Visual summaries of GRI and other CGM metrics could accelerate both risk assessment and the evaluation of therapeutic responses (32).

Baseline characteristics such as female sex, longer diabetes duration, lower HbA1c, and more hypoglycaemia were associated with improved GRI, while behavioural and psychological factors also played a role. For example, greater reductions in hypoglycaemia risk when switching to CGM were observed in individuals describing themselves as thorough, while distractibility and self-described laziness were linked to smaller improvements. A slight increase in hypoglycaemia risk among those trained in carbohydrate counting may reflect greater clinical complexity or overconfidence in insulin dosing. Higher fear of hypoglycaemia at baseline was associated with improvements in hyperglycaemia risk, suggesting that CGM may provide reassurance and support more balanced glucose management in this group. These exploratory findings indicate that individual traits may influence CGM effectiveness. However, the lack of consistent associations between changes in GRI and general participant-reported outcomes such as well-being or quality of life indicates that objective improvements in glycaemic control may not directly translate into perceived improvements in daily life. This echoes previous findings in the GOLD trial (18), and is an area that warrants further investigation.

GRI may serve as a valuable single metric for summarising glycaemic quality in clinical care, complementing HbA1c and TIR. Its distinct hypo- and hyperglycaemia components, together with visual representation, provide actionable insights into underlying glucose patterns. The greater sensitivity to certain treatment effects compared with TIR suggests that GRI could be particularly useful for identifying patients most likely to benefit from CGM, especially those at risk of both glycaemic extremes. It may also be suitable as an endpoint in certain clinical trials to capture effects on various glucose patterns that TIR alone might miss. Given the relatively low baseline exposure to hypoglycaemia in this cohort, the potential utility of GRI may be even greater in populations with problematic hypoglycaemia, where reducing extreme low-glucose events is a key clinical goal. However, in the clinical management of T1D, conventional CGM metrics such as TIR, TBR, and TAR remain highly valuable because of their simplicity and ease of interpretation, as they directly express the percentage of time spent in different glucose ranges. Further, for health professionals working with diabetes, a relatively good overview can often be achieved using only a few key metrics such as TIR in combination with time in hypoglycaemia. Thus, while GRI may have several potential applications, conventional CGM metrics remain essential for everyday clinical decision-making. Further research on the relationship between GRI and diabetes complications is essential, particularly if specific GRI levels are used as key metrics. This knowledge is critical for understanding how complication incidence is associated with GRI levels when monitoring patients in clinical practice.

Key strengths of this study include the randomised crossover design, within-subject comparisons, and standardised assessments where all participants used the same CGM device, central laboratory for HbA1c measurement, and procedures for collecting research data. The GRI may be particularly valuable in primary care, where familiarity with multiple CGM metrics may be limited, as it provides a single summary measure of glycaemic quality.

Limitations of this study included the exploratory nature of the psychosocial associations, the lack of validated clinical cut-off points for GRI, the restriction of the study population to adults with moderately elevated HbA1c receiving MDI therapy, and the absence of data on long-term diabetes complications.

## 5 Conclusions

Switching from SMBG to CGM significantly reduced the GRI in adults with T1D on MDI therapy, with improvements in both the hypo- and hyperglycaemia components. GRI correlated moderately to strongly with established CGM metrics, and the observed effect sizes were larger than for TIR in this dataset. As a composite metric that integrates both hypo- and hyperglycaemia, GRI may serve as a valuable endpoint for intervention studies and as a complementary tool in clinical practice. Further research is needed to validate its clinical utility and generalisability across broader populations and relation to diabetes complications.

## Data Availability

The datasets presented in this article are not readily available because of ethical restrictions. However, data may be provided upon reasonable request for academic purposes. Requests to access the datasets should be directed to Marcus Lind, marcus.lind@gu.se.
