# Supplementary material for: Impact of continuous glucose monitoring on glycaemic risk index in adults with type 1 diabetes using multiple daily insulin injections in the GOLD trial
Source: Front Clin Diabetes Healthc. 2026 Mar 4;7:1767987. doi: 10.3389/fcdhc.2026.1767987 (PMC12995639; doi:10.3389/fcdhc.2026.1767987)
Supplement: Supplementary file 1 [file DataSheet1.docx]

**Supplemental Table S1.** Correlations between changes in Glycaemia Risk Index (GRI), its hypoglycaemia and hyperglycaemia components, and changes in CGM-derived metrics during continuous glucose monitoring (CGM) compared to self-monitoring of blood glucose (SMBG).

| CGM metric | GRI, unadjusted | *P* | GRI, adjusted * | *P* | Hypoglycaemia component,  unadjusted | *P* | Hypoglycaemia component,  adjusted * | *P* | Hyperglycaemia component, unadjusted | *P* | Hyperglycaemia component,  adjusted * | *P* |
| --- | --- | --- | --- | --- | --- | --- | --- | --- | --- | --- | --- | --- |
| Mean glucose level | 0.65 (0.54, 0.74) | <.001 | 0.65 (0.52, 0.75) | <.001 | −0.58 (−0.68, −0.44) | <.001 | −0.55 (−0.67, −0.39) | <.001 | 0.90 (0.86, 0.93) | <.001 | 0.89 (0.84, 0.93) | <.001 |
| TBR level 2 (<3.0 mmol/L) | 0.27 (0.10, 0.43) | 0.002 | 0.26 (0.07, 0.43) | 0.009 | 0.92 (0.88, 0.94) | <.001 | 0.89 (0.84, 0.93) | <.001 | −0.19 (−0.36, −0.01) | 0.034 | −0.16 (−0.35, 0.03) | 0.098 |
| TBR (<3.9 mmol/L) | 0.11 (−0.07, 0.28) | 0.24 | 0.09 (−0.11, 0.28) | 0.38 | 1.00 (1.00, 1.00) | <.001 | 1.00 (1.00, 1.00) | <.001 | −0.39 (−0.53, −0.22) | <.001 | −0.37 (−0.53, −0.19) | <.001 |
| TIR (3.9–10.0 mmol/L) | −0.47 (−0.60, −0.32) | <.001 | −0.46 (−0.60, −0.29) | <.001 | 0.44 (0.28, 0.57) | <.001 | 0.43 (0.26, 0.58) | <.001 | −0.66 (−0.75, −0.54) | <.001 | −0.64 (−0.74, −0.51) | <.001 |
| TAR (>10.0 mmol/L) | 0.85 (0.79, 0.89) | <.001 | 0.85 (0.78, 0.89) | <.001 | −0.36 (−0.50, −0.19) | <.001 | −0.38 (−0.53, −0.20) | <.001 | 0.97 (0.96, 0.98) | <.001 | 0.97 (0.96, 0.98) | <.001 |
| TAR level 2 (>13.9 mmol/L) | 0.83 (0.76, 0.87) | <.001 | 0.83 (0.76, 0.88) | <.001 | −0.35 (−0.50, −0.18) | <.001 | −0.31 (−0.48, −0.12) | <.001 | 0.96 (0.94, 0.97) | <.001 | 0.96 (0.94, 0.97) | <.001 |
| SD of glucose values | 0.55 (0.41, 0.66) | <.001 | 0.53 (0.39, 0.65) | <.001 | 0.02 (−0.15, 0.20) | 0.80 | 0.04 (−0.14, 0.22) | 0.64 | 0.48 (0.33, 0.60) | <.001 | 0.45 (0.30, 0.59) | <.001 |
| Pearson correlation coefficients are presented with 95% confidence intervals (CIs).  Positive values indicate that increases in the listed CGM metrics are associated with smaller reductions in GRI or its components during the transition from SMBG to CGM, whereas negative values indicate that increases in CGM metrics are associated with greater reductions.  * Adjusted for GRI, hypoglycaemia component, and hyperglycaemia component baseline values, respectively.  **Abbreviations**: CI, Confidence Interval; SD, Standard Deviation; TAR, Time Above Range; TBR, Time Below Range; TIR, Time in Range. | | | | | | | | | | | | |

**Supplemental Table S2.** Correlations between baseline variables from the PredQ questionnaire and changes in Glycaemia Risk Index (GRI), hypoglycaemia component, and hyperglycaemia component during continuous glucose monitoring (CGM) compared to self-monitoring of blood glucose (SMBG).

| Baseline variable | GRI, unadjusted | *P* | GRI, adjusted * | *P* | Hypoglycaemia component,  unadjusted | *P* | Hypoglycaemia component,  adjusted * | *P* | Hyperglycaemia component,  unadjusted | *P* | Hyperglycaemia component,  adjusted * | *P* |
| --- | --- | --- | --- | --- | --- | --- | --- | --- | --- | --- | --- | --- |
| Work-related |  |  |  |  |  |  |  |  |  |  |  |  |
| Educational level | −0.13 (−0.30, 0.05) | 0.16 | −0.08 (−0.28, 0.12) | 0.44 | 0.03 (−0.15, 0.21) | 0.72 | 0.01 (−0.20, 0.21) | 0.94 | −0.14 (−0.31, 0.04) | 0.13 | −0.09 (−0.28, 0.12) | 0.41 |
| Employed | −0.06 (−0.24, 0.11) | 0.48 | NA | NA | 0.07 (−0.11, 0.25) | 0.42 | NA | NA | −0.09 (−0.26, 0.09) | 0.34 | NA | NA |
| Working hours | −0.04 (−0.24, 0.16) | 0.68 | −0.08 (−0.28, 0.12) | 0.43 | −0.07 (−0.26, 0.13) | 0.49 | −0.06 (−0.26, 0.15) | 0.60 | 0.00 (−0.20, 0.20) | 1.00 | −0.04 (−0.24, 0.16) | 0.70 |
| Retired | 0.06 (−0.12, 0.23) | 0.54 | NA | NA | −0.07 (−0.24, 0.11) | 0.45 | NA | NA | 0.09 (−0.09, 0.26) | 0.35 | NA | NA |
| Sickness benefit | −0.08 (−0.25, 0.10) | 0.38 | −0.10 (−0.30, 0.11) | 0.34 | −0.04 (−0.22, 0.14) | 0.65 | −0.11 (−0.31, 0.10) | 0.29 | −0.05 (−0.23, 0.13) | 0.58 | −0.04 (−0.24, 0.16) | 0.69 |
| Student | −0.09 (−0.26, 0.09) | 0.33 | −0.09 (−0.29, 0.12) | 0.41 | 0.02 (−0.16, 0.20) | 0.81 | 0.07 (−0.14, 0.27) | 0.52 | −0.09 (−0.26, 0.09) | 0.33 | −0.10 (−0.30, 0.11) | 0.34 |
| Unemployed | −0.08 (−0.26, 0.09) | 0.35 | −0.04 (−0.24, 0.17) | 0.74 | −0.07 (−0.24, 0.11) | 0.47 | −0.06 (−0.26, 0.15) | 0.59 | −0.06 (−0.24, 0.11) | 0.48 | 0.00 (−0.20, 0.21) | 0.96 |
| Sick leave | 0.05 (−0.13, 0.23) | 0.57 | −0.03 (−0.23, 0.18) | 0.81 | −0.06 (−0.23, 0.12) | 0.54 | −0.17 (−0.36, 0.04) | 0.11 | 0.09 (−0.09, 0.26) | 0.32 | 0.05 (−0.16, 0.25) | 0.64 |
|  |  |  |  |  |  |  |  |  |  |  |  |  |
| Diabetes-related |  |  |  |  |  |  |  |  |  |  |  |  |
| Carbohydrate counting | 0.01 (−0.17, 0.19) | 0.88 | 0.03 (−0.18, 0.23) | 0.80 | 0.14 (−0.04, 0.31) | 0.13 | 0.09 (−0.11, 0.29) | 0.37 | −0.05 (−0.23, 0.13) | 0.59 | −0.03 (−0.23, 0.18) | 0.81 |
| Trained in carbohydrate counting | −0.07 (−0.25, 0.11) | 0.43 | 0.03 (−0.17, 0.23) | 0.76 | 0.19 (0.01, 0.35) | 0.041 | 0.11 (−0.10, 0.31) | 0.29 | −0.16 (−0.33, 0.02) | 0.082 | −0.01 (−0.21, 0.20) | 0.94 |
| Improve glucose control | −0.09 (−0.26, 0.09) | 0.35 | −0.06 (−0.26, 0.14) | 0.55 | −0.00 (−0.18, 0.17) | 0.97 | −0.00 (−0.21, 0.20) | 0.96 | −0.08 (−0.25, 0.10) | 0.39 | −0.05 (−0.25, 0.15) | 0.62 |
| Improve HbA1c | −0.08 (−0.25, 0.10) | 0.40 | −0.08 (−0.27, 0.13) | 0.47 | 0.00 (−0.18, 0.18) | 1.00 | 0.04 (−0.17, 0.24) | 0.73 | −0.08 (−0.25, 0.10) | 0.39 | −0.10 (−0.30, 0.11) | 0.34 |
| Avoid low blood sugar levels | 0.13 (−0.05, 0.30) | 0.17 | 0.12 (−0.09, 0.32) | 0.25 | −0.10 (−0.27, 0.08) | 0.27 | −0.20 (−0.38, 0.01) | 0.060 | 0.16 (−0.02, 0.32) | 0.086 | 0.17 (−0.03, 0.36) | 0.10 |
| Avoid high blood sugar levels | −0.03 (−0.20, 0.15) | 0.78 | −0.06 (−0.26, 0.15) | 0.56 | −0.07 (−0.25, 0.11) | 0.42 | −0.14 (−0.33, 0.07) | 0.18 | 0.00 (−0.18, 0.18) | 0.99 | −0.01 (−0.22, 0.19) | 0.91 |
| Improved quality of life | 0.04 (−0.14, 0.21) | 0.70 | 0.11 (−0.10, 0.30) | 0.31 | 0.03 (−0.14, 0.21) | 0.71 | 0.07 (−0.14, 0.27) | 0.52 | 0.01 (−0.17, 0.19) | 0.93 | 0.06 (−0.15, 0.26) | 0.59 |
|  |  |  |  |  |  |  |  |  |  |  |  |  |
| Personality-related (I am): |  |  |  |  |  |  |  |  |  |  |  |  |
| Thorough | 0.01 (−0.17, 0.19) | 0.89 | 0.00 (−0.20, 0.20) | 1.00 | −0.20 (−0.36, −0.02) | 0.030 | −0.19 (−0.38, 0.02) | 0.069 | 0.09 (−0.09, 0.27) | 0.31 | 0.07 (−0.13, 0.27) | 0.49 |
| Efficient | −0.07 (−0.25, 0.11) | 0.42 | −0.04 (−0.25, 0.16) | 0.68 | −0.06 (−0.24, 0.12) | 0.50 | −0.00 (−0.21, 0.20) | 0.98 | −0.04 (−0.22, 0.14) | 0.65 | −0.05 (−0.25, 0.16) | 0.65 |
| Ambitious | 0.01 (−0.17, 0.19) | 0.89 | −0.02 (−0.22, 0.19) | 0.88 | −0.10 (−0.28, 0.08) | 0.26 | −0.04 (−0.24, 0.17) | 0.71 | 0.07 (−0.11, 0.25) | 0.44 | 0.00 (−0.20, 0.21) | 0.97 |
| Reliable | 0.05 (−0.13, 0.23) | 0.58 | 0.16 (−0.04, 0.35) | 0.12 | −0.12 (−0.29, 0.06) | 0.19 | −0.07 (−0.27, 0.14) | 0.53 | 0.10 (−0.08, 0.27) | 0.30 | 0.16 (−0.04, 0.35) | 0.12 |
| Persistent | −0.02 (−0.20, 0.16) | 0.83 | −0.03 (−0.23, 0.18) | 0.78 | −0.09 (−0.26, 0.09) | 0.35 | −0.06 (−0.26, 0.15) | 0.59 | 0.01 (−0.17, 0.18) | 0.95 | −0.02 (−0.22, 0.19) | 0.87 |
| Easily distracted | 0.14 (−0.04, 0.31) | 0.14 | 0.16 (−0.04, 0.35) | 0.12 | 0.21 (0.03, 0.37) | 0.024 | 0.13 (−0.08, 0.33) | 0.21 | 0.03 (−0.15, 0.21) | 0.71 | 0.09 (−0.11, 0.29) | 0.37 |
| Careless | 0.01 (−0.17, 0.19) | 0.92 | 0.07 (−0.14, 0.27) | 0.53 | 0.04 (−0.14, 0.22) | 0.65 | −0.10 (−0.30, 0.11) | 0.35 | −0.01 (−0.19, 0.17) | 0.92 | 0.11 (−0.09, 0.31) | 0.29 |
| Lazy | −0.08 (−0.26, 0.10) | 0.37 | −0.07 (−0.27, 0.13) | 0.48 | 0.27 (0.09, 0.43) | 0.003 | 0.22 (0.02, 0.41) | 0.032 | −0.21 (−0.37, −0.03) | 0.021 | −0.17 (−0.36, 0.03) | 0.098 |
| Organised | −0.03 (−0.21, 0.15) | 0.76 | −0.03 (−0.23, 0.18) | 0.80 | −0.03 (−0.20, 0.15) | 0.78 | −0.15 (−0.34, 0.06) | 0.16 | −0.01 (−0.19, 0.17) | 0.90 | 0.05 (−0.16, 0.25) | 0.65 |
| The PredQ (Predictors Questionnaire) includes work-related, diabetes-related, and personality-related items assessed at baseline to explore factors potentially associated with changes in glycaemic control. Higher scores reflect greater agreement or identification with the stated attribute or belief.  Pearson correlation coefficients with 95% confidence intervals (CIs) and corresponding *P* values are shown.  Positive values indicate that higher levels of the baseline variable are associated with smaller reductions in GRI or its components during the transition from SMBG to CGM, whereas negative values indicate that higher baseline levels are associated with greater reductions.  * Adjusted for GRI, hypoglycaemia component, and hyperglycaemia component baseline values, respectively.  **Abbreviations**: CI, Confidence Interval; NA, not estimable due to small numbers in the unemployed and retired groups. | | | | | | | | | | | | |

**Supplemental Table S3.** Correlations between baseline variables and changes in Glycaemia Risk Index (GRI), its hypoglycaemia and hyperglycaemia components, during continuous glucose monitoring (CGM) compared to self-monitoring of blood glucose (SMBG).

| Baseline characteristic | GRI change, unadjusted | *P* | GRI change, adjusted * | *P* | Hypoglycaemia component change, unadjusted | *P* | Hypoglycaemia component change, adjusted * | *P* | Hyperglycaemia component change, unadjusted | *P* | Hyperglycaemia component change, adjusted * | *P* |
| --- | --- | --- | --- | --- | --- | --- | --- | --- | --- | --- | --- | --- |
| Age | 0.10 (−0.08, 0.27) | 0.28 | 0.07 (−0.12, 0.26) | 0.46 | −0.03 (−0.21, 0.15) | 0.74 | −0.10 (−0.28, 0.09) | 0.32 | 0.11 (−0.06, 0.29) | 0.21 | 0.11 (−0.08, 0.30) | 0.24 |
| Female sex | 0.06 (−0.12, 0.24) | 0.49 | 0.06 (−0.13, 0.24) | 0.55 | −0.21 (−0.37, −0.03) | 0.020 | −0.14 (−0.32, 0.05) | 0.16 | 0.14 (−0.04, 0.31) | 0.11 | 0.11 (−0.08, 0.29) | 0.26 |
| HbA1c | 0.06 (−0.12, 0.24) | 0.49 | 0.11 (−0.08, 0.29) | 0.25 | 0.21 (0.03, 0.37) | 0.021 | −0.02 (−0.20, 0.17) | 0.87 | −0.05 (−0.22, 0.13) | 0.60 | 0.06 (−0.13, 0.25) | 0.53 |
| Diabetes duration | −0.03 (−0.21, 0.15) | 0.73 | −0.09 (−0.28, 0.10) | 0.33 | −0.29 (−0.44, −0.11) | 0.001 | −0.27 (−0.43, −0.08) | 0.005 | 0.10 (−0.08, 0.27) | 0.26 | 0.05 (−0.14, 0.24) | 0.60 |
| No. hypoglycaemias past two months | 0.10 (−0.08, 0.28) | 0.27 | 0.12 (−0.07, 0.30) | 0.22 | 0.13 (−0.06, 0.30) | 0.18 | 0.08 (−0.11, 0.27) | 0.40 | 0.03 (−0.15, 0.21) | 0.74 | 0.06 (−0.14, 0.24) | 0.57 |
| No. severe hypoglycaemias past year | 0.11 (−0.07, 0.28) | 0.23 | 0.12 (−0.07, 0.31) | 0.20 | −0.08 (−0.25, 0.10) | 0.39 | −0.03 (−0.21, 0.16) | 0.79 | 0.17 (−0.01, 0.34) | 0.060 | 0.18 (−0.01, 0.35) | 0.065 |
| No. severe hypoglycaemias past five yrs | 0.04 (−0.14, 0.22) | 0.66 | 0.05 (−0.14, 0.24) | 0.58 | −0.03 (−0.21, 0.14) | 0.70 | 0.02 (−0.17, 0.21) | 0.81 | 0.07 (−0.11, 0.24) | 0.43 | 0.07 (−0.12, 0.25) | 0.48 |
| Mean glucose level | 0.01 (−0.17, 0.19) | 0.90 | 0.06 (−0.13, 0.25) | 0.53 | 0.23 (0.06, 0.39) | 0.009 | −0.05 (−0.23, 0.14) | 0.64 | −0.09 (−0.27, 0.08) | 0.30 | 0.02 (−0.17, 0.21) | 0.83 |
| TBR (<3.9 mmol/L) | −0.13 (−0.30, 0.05) | 0.17 | −0.07 (−0.25, 0.12) | 0.48 | −0.41 (−0.55, −0.25) | <.001 | −0.10 (−0.28, 0.09) | 0.32 | 0.08 (−0.10, 0.26) | 0.37 | 0.06 (−0.13, 0.25) | 0.52 |
| TBR level 2 (<3.0 mmol/L) | −0.13 (−0.30, 0.05) | 0.15 | −0.08 (−0.26, 0.11) | 0.41 | −0.44 (−0.57, −0.28) | <.001 | 0.10 (−0.09, 0.28) | 0.32 | 0.09 (−0.09, 0.26) | 0.34 | 0.04 (−0.15, 0.23) | 0.67 |
| TIR (3.9–10.0 mmol/L) | −0.03 (−0.21, 0.15) | 0.77 | −0.09 (−0.27, 0.10) | 0.37 | −0.15 (−0.32, 0.03) | 0.11 | −0.01 (−0.20, 0.18) | 0.94 | 0.04 (−0.14, 0.22) | 0.63 | −0.04 (−0.22, 0.15) | 0.70 |
| TAR (>10.0 mmol/L) | −0.08 (−0.25, 0.11) | 0.41 | 0.05 (−0.14, 0.23) | 0.63 | 0.21 (0.03, 0.37) | 0.022 | 0.02 (−0.17, 0.21) | 0.86 | −0.16 (−0.33, 0.02) | 0.073 | −0.06 (−0.25, 0.13) | 0.52 |
| TAR level 2 (>13.9 mmol/L) | −0.10 (−0.27, 0.08) | 0.28 | 0.03 (−0.16, 0.22) | 0.77 | 0.11 (−0.07, 0.29) | 0.22 | −0.05 (−0.24, 0.14) | 0.58 | −0.14 (−0.31, 0.04) | 0.13 | 0.06 (−0.13, 0.25) | 0.52 |
| DTSQs | 0.16 (−0.02, 0.33) | 0.082 | 0.18 (−0.01, 0.35) | 0.068 | −0.03 (−0.21, 0.15) | 0.74 | −0.02 (−0.21, 0.17) | 0.85 | 0.16 (−0.02, 0.33) | 0.078 | 0.17 (−0.02, 0.35) | 0.072 |
| WHO-5 | −0.03 (−0.21, 0.15) | 0.73 | −0.04 (−0.22, 0.15) | 0.71 | −0.10 (−0.27, 0.08) | 0.28 | −0.01 (−0.20, 0.18) | 0.89 | 0.03 (−0.15, 0.20) | 0.78 | 0.00 (−0.19, 0.19) | 0.99 |
| Swe-PAID-20 | 0.08 (−0.10, 0.25) | 0.40 | 0.09 (−0.10, 0.28) | 0.33 | 0.17 (−0.01, 0.33) | 0.066 | 0.07 (−0.12, 0.25) | 0.48 | −0.01 (−0.19, 0.16) | 0.88 | 0.02 (−0.17, 0.21) | 0.81 |
| HCS | −0.13 (−0.30, 0.06) | 0.17 | −0.13 (−0.31, 0.06) | 0.19 | −0.24 (−0.40, −0.06) | 0.010 | −0.20 (−0.38, −0.01) | 0.035 | −0.01 (−0.19, 0.17) | 0.88 | −0.02 (−0.21, 0.17) | 0.82 |
| Swe-HFS: Behaviour/Avoidance | 0.20 (0.03, 0.37) | 0.023 | 0.20 (0.01, 0.37) | 0.042 | 0.00 (−0.18, 0.18) | 0.99 | −0.07 (−0.25, 0.12) | 0.49 | 0.20 (0.02, 0.37) | 0.026 | 0.21 (0.02, 0.38) | 0.029 |
| Swe-HFS: Worry | 0.16 (−0.02, 0.33) | 0.078 | 0.19 (0.00, 0.37) | 0.047 | 0.10 (−0.08, 0.27) | 0.28 | 0.10 (−0.10, 0.28) | 0.33 | 0.11 (−0.07, 0.29) | 0.21 | 0.14 (−0.06, 0.32) | 0.16 |
| Pearson correlation coefficients are presented with 95% confidence intervals (CIs).  Positive values indicate that higher levels of the baseline variable are associated with smaller reductions in GRI or its components during the transition from SMBG to CGM, whereas negative values indicate that higher baseline levels are associated with greater reductions.  DTSQs, WHO-5, HCS: higher scores indicate better outcomes (higher satisfaction, well-being, confidence). Swe-PAID-20 and Swe-HFS: higher scores indicate worse outcomes (greater distress or fear).  * Adjusted for GRI, hypoglycaemia component, and hyperglycaemia component baseline values, respectively.  **Abbreviations:** CI, Confidence Interval; DTSQs, Diabetes Treatment Satisfaction Questionnaire, Status Version; HCS, Hypoglycaemic Confidence Scale; HFS, Hypoglycaemic Fear Survey; PAID-20, Problem Areas in Diabetes Scale; Swe, Swedish Version; TAR, Time Above Range; TBR, Time Below Range; TIR, Time in Range; WHO, World Health Organization. | | | | | | | | | | | | |

**Supplemental Table S4.** Correlations between changes in Glycaemia Risk Index (GRI), its hypoglycaemia and hyperglycaemia components, and changes in participant-reported outcomes (PROs) during continuous glucose monitoring (CGM) compared to self-monitoring of blood glucose (SMBG).

| Participant-reported outcome | GRI, unadjusted | *P* | GRI, adjusted * | *P* | Hypoglycaemia component,  unadjusted | *P* | Hypoglycaemia component,  adjusted * | *P* | Hyperglycaemia component, unadjusted | *P* | Hyperglycaemia component,  adjusted * | *P* |
| --- | --- | --- | --- | --- | --- | --- | --- | --- | --- | --- | --- | --- |
| DTSQs | −0.14 (−0.32, 0.04) | 0.13 | −0.16 (−0.34, 0.04) | 0.11 | 0.03 (−0.15, 0.22) | 0.72 | −0.08 (−0.27, 0.11) | 0.40 | −0.14 (−0.31, 0.05) | 0.14 | −0.13 (−0.31, 0.07) | 0.20 |
| DTSQc | −0.01 (−0.19, 0.17) | 0.94 | −0.00 (−0.20, 0.19) | 0.98 | 0.03 (−0.15, 0.21) | 0.73 | 0.09 (−0.11, 0.27) | 0.39 | −0.03 (−0.21, 0.15) | 0.74 | −0.04 (−0.23, 0.15) | 0.67 |
| WHO-5 | −0.10 (−0.27, 0.08) | 0.27 | −0.10 (−0.29, 0.09) | 0.30 | 0.13 (−0.05, 0.30) | 0.16 | 0.10 (−0.09, 0.29) | 0.30 | −0.15 (−0.32, 0.03) | 0.11 | −0.15 (−0.33, 0.05) | 0.14 |
| Swe-PAID-20 | 0.07 (−0.11, 0.24) | 0.45 | 0.06 (−0.13, 0.25) | 0.54 | −0.06 (−0.23, 0.12) | 0.54 | −0.06 (−0.25, 0.14) | 0.58 | 0.08 (−0.10, 0.25) | 0.41 | 0.06 (−0.13, 0.25) | 0.53 |
| HCS | −0.01 (−0.19, 0.17) | 0.92 | 0.03 (−0.17, 0.22) | 0.79 | −0.01 (−0.19, 0.17) | 0.95 | −0.07 (−0.26, 0.13) | 0.50 | −0.01 (−0.19, 0.17) | 0.93 | 0.04 (−0.16, 0.23) | 0.71 |
| Swe-HFS: Behaviour/Avoidance | 0.03 (−0.15, 0.20) | 0.78 | 0.03 (−0.17, 0.22) | 0.80 | 0.15 (−0.03, 0.31) | 0.11 | 0.17 (−0.03, 0.35) | 0.088 | −0.05 (−0.23, 0.13) | 0.57 | −0.06 (−0.25, 0.14) | 0.55 |
| Swe-HFS: Worry | 0.07 (−0.11, 0.24) | 0.44 | 0.09 (−0.10, 0.28) | 0.35 | −0.15 (−0.31, 0.03) | 0.11 | −0.06 (−0.25, 0.14) | 0.56 | 0.14 (−0.04, 0.31) | 0.11 | 0.14 (−0.06, 0.32) | 0.17 |
| Pearson correlation coefficients are presented with 95% confidence intervals (CIs).  Positive values indicate that increases in the listed participant-reported outcomes are associated with smaller reductions in GRI or its components during the transition from SMBG to CGM, whereas negative values indicate that increases in participant-reported outcomes are associated with greater reductions.  DTSQs, WHO-5, HCS: higher scores indicate better outcomes (higher satisfaction, well-being, confidence). Swe-PAID-20 and Swe-HFS: higher scores indicate worse outcomes (greater distress or fear).  * Adjusted for GRI, hypoglycaemia component, and hyperglycaemia component baseline values, respectively.  **Abbreviations**: CI, Confidence Interval; DTSQc, Diabetes Treatment Satisfaction Questionnaire, Change Version; DTSQs, Diabetes Treatment Satisfaction Questionnaire, Status Version; HCS, Hypoglycaemic Confidence Scale; HFS, Hypoglycaemic Fear Survey; PAID-20, Problem Areas in Diabetes Scale; Swe, Swedish Version; WHO-5, World Health Organization–Five Well-Being Index. | | | | | | | | | | | | |
